# Systematic mapping of *O*-GlcNAc transferase and *O*-GlcNAcase defines disease-associated variants

**DOI:** 10.1016/j.jbc.2026.113134

**Published:** 2026-05-08

**Authors:** Jonah Kimi, Florian Malard, Stephanie Olivier-Van Stichelen

**Affiliations:** 1 Department of Biochemistry, Medical College of Wisconsin, Milwaukee, Wisconsin, USA; 2 Univ. Bordeaux, CNRS, INSERM, ARNA, UMR 5320, U1212, Bordeaux, France; 3 Department of Neurosurgery, Medical College of Wisconsin, Milwaukee, Wisconsin, USA; 4 Department of Obstetrics & Gynecology, Medical College of Wisconsin, Milwaukee, Wisconsin, USA; 5 Data Science Institute, Medical College of Wisconsin, Milwaukee, Wisconsin, USA

**Keywords:** cancer, database, intellectual disability, O-GlcNAc transferase, O-GlcNAcase, OGT-XLID, variant

## Abstract

For decades, *O-*GlcNAcylation has been recognized as a critical posttranslational modification involved in numerous physiological processes and increasingly implicated in human disease. Despite substantial evidence linking *O*-GlcNAcylation to neurodegeneration and cancer, *O*-GlcNAc cycling enzymes were long considered so essential that any meaningful amino acid substitution would not be tolerated in humans. However, advances in genetic screening have recently identified viable single-nucleotide variants (SNVs) in *O*-GlcNAc Transferase (OGT) in individuals with X-linked intellectual disability (OGT-XLID). The growing identification of affected families prompted a reevaluation of how subtle genomic variation in O-GlcNAc enzymes contributes to human pathology. Here, we present the first comprehensive catalog of variants in both *OGT* (oglcnac.mcw.edu/ogtoga/ogt/) and *O*-GlcNAcase (*OGA*) (oglcnac.mcw.edu/ogtoga/oga/), the two enzymes that regulate *O*-GlcNAcylation. This resource integrates cancer-associated mutations, population allele frequencies, and structural mapping onto both protein structures. Recognizing that public repositories such as ClinVar and gnomAD capture only a portion of clinically relevant variation, we partnered directly with clinicians and researchers to curate the most comprehensive and up-to-date collection of pathogenic OGT-XLID variants (n = 101). By combining population datasets with cancer mutation databases, we identify distinct hotspot mutations with opposing clinical associations: *OGT* hotspot mutations correlate with improved survival in cancer patients, whereas *OGA* hotspot mutations are associated with reduced overall survival. Together, this resource establishes a framework for understanding genotype–phenotype relationships in *O*-GlcNAc biology and provides a foundation for future mechanistic, translational, and clinical investigations.

*O*-GlcNAcylation has remained one of the most underappreciated post-translational modifications of the past three decades, despite its broad impact on cell signaling, metabolism, and disease. *O*-GlcNAcylation is the addition of a single N-acetylglucosamine moiety to serine and threonine residues, which appears simple, yet key aspects of its homeostasis and regulation remain poorly understood (1). Although often compared to phosphorylation, *O*-GlcNAcylation differs fundamentally. Rather than being regulated by dozens of kinases and phosphatases, it is controlled solely by one transferase (OGT) and one hydrolase (OGA) (Fig. 1*A*). How these two enzymes achieve substrate and site specificity at the right time and in the right context remains a central unanswered question in the field. The absence of a consensus sequence, combined with the lack of fully accurate prediction tools, continues to limit our understanding of the full regulatory landscape of *O*-GlcNAcylation.

**Figure 1:**
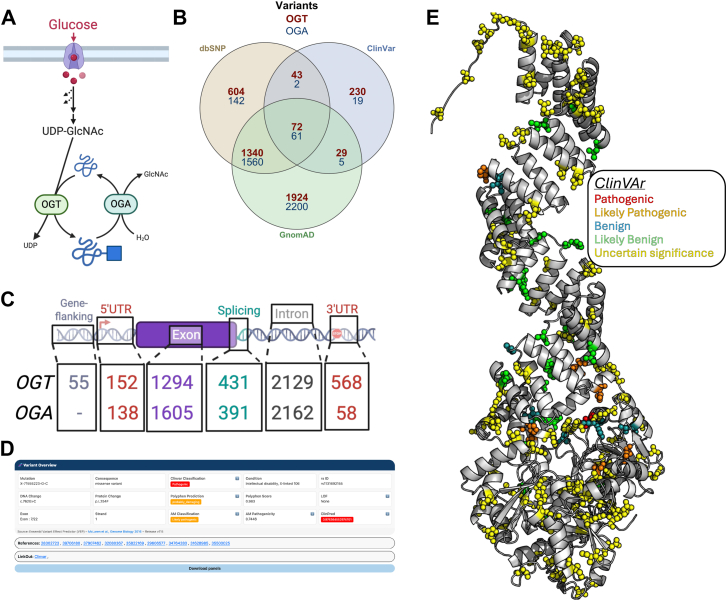
**Overview of *OGT* and *OGA* variant integration, annotation, and visualization in the *O*-GlcNAc Database.***A*, schematic representation of the *O*-GlcNAc cycling pathway, illustrating the opposing enzymatic activities of *O*-GlcNAc transferase (OGT) and *O*-GlcNAcase (OGA) in regulating protein *O*-GlcNAcylation in response to glucose-derived UDP-GlcNAc availability. *B*, Venn diagram showing the overlap of *OGT* and *OGA* variants retrieved from dbSNP, ClinVar, and gnomAD. Variant counts are shown separately for *OGT* (*red*) and *OGA* (*blue*) within each dataset and intersection. *C*, Genomic distribution of *OGT* and *OGA* single-nucleotide variants across annotated genomic regions, including gene-flanking regions, 5′ untranslated regions (5′UTR), exons, splice junctions, introns, and 3′ untranslated regions (3′UTR). *D*, screenshot of the variant overview page within the *O*-GlcNAc Database. Each individual variant displays genomic coordinates, predicted functional impact, pathogenicity classifications, allele frequency information, cancer associations, and links to external resources. *E*, three-dimensional structural mapping of ClinVar-annotated variants onto a predicted structure model (AlphaFold) of human OGT (AF-O15294-F1). Variants are displayed as spheres on the protein backbone and colored according to ClinVar classification.

To address these challenges, we established the *O*-GlcNAc Database in 2020 (oglcnac.mcw.edu), which compiles experimentally validated proteins and modification sites (2). In this resource, we currently catalog over 24,021 proteins and 24,940 sites across 65 species with detailed literature curation, biological context annotation, a quantitative *O*-GlcNAc score to assess data reliability, and structural visualization of *O*-GlcNAc sites onto proteins (3, 4). In addition, the database provides advanced tools such as an ontology framework and CytOVS for the analysis of *O-*GlcNAcome datasets derived from mass spectrometry studies (5). Since its release, the *O*-GlcNAc database has served as a shared resource for the research community, encouraging both newcomers and established investigators to explore this essential, highly dynamic modification in greater depth.

Building on this foundation, we now extend this effort to establish the first comprehensive catalog of *OGT* and *OGA* variants. Indeed, another important milestone in the *O*-GlcNAc field was the identification of the first human *OGT* point mutations associated with X-linked intellectual disability (XLID) (6, 7). Until then, it was widely assumed that *O*-GlcNAc mutations were not viable in humans and that OGT was so essential that only intact, fully functioning OGT proteins were present. Indeed, all attempts to delete *OGT* or *OGA* resulted in early lethality in mice, suggesting that the absence of OGT or its nonfunctional form is nonviable (8). Thanks to the advances in genetic screening, the first patient was identified in 2015 with a single point mutation on the *OGT* gene, leading to pathogenic presentation of X-linked intellectual disabilities (6). Since then, more OGT-XLID variants have been identified, and the list is growing thanks to patient and clinician communities that raised awareness around this condition (9, 10, 11, 12, 13, 14, 15).

Overall, the discovery of these patients prompted the field to start investigating the molecular consequences of these mutations and, surprisingly, observed that some of the variants were not causing a major defect in *O*-GlcNAcylation levels, nor were they impacting the cleavage of HCF1, despite this being the main functions described for OGT (9, 11, 12, 14, 16, 17). Once more, these findings reveal a major gap in our mechanistic understanding, suggesting that OGT mutation clearly leads to symptomatic patients with XLID without critically impacting its known functions or transcript/protein expression (12, 14).

Structural and biochemical studies further suggest that OGT catalysis proceeds through an ordered bi-bi mechanism, in which UDP-GlcNAc binding precedes peptide substrate engagement, with contributions from both enzyme residues and the substrate itself to facilitate glycosyl transfer (18). Prior efforts to investigate the importance of specific amino acids in the OGT structure identified K842 as the key residue for OGT catalytic activity, including both *O*-GlcNAcylation and HCF1 cleavage (17). On the other hand, D554 has been identified as key for HCF1 cleavage but not *O*-GlcNAcylation, while substitution of 5 asparagine residues 322, 356, 390, 424, 458 to alanine leads to the loss of HCF1 cleavage activity (17). Indeed, the other key domain for OGT is the tetratricopeptide repeat (TPR) domain. The TPRs form a superhelical scaffold that mediates substrate recognition through a conserved asparagine ladder, enabling backbone-dependent interactions and contributing to OGT’s broad but selective substrate repertoire (19). Interestingly, OGT-XLID variants in these TPR domains alter OGT expression, thermostability, its interactome, and HCF1 cleavage (11, 13, 20). Similarly, efforts to identify specific differences in the TPR interaction domain of OGT identified distinct interacting proteins for each TPR and suggested that variants in each TPR can lead to subtle changes in the OGT interactome (20, 21).

Overall, for OGA, the function of specific residues has not been well defined, partly due to the delayed resolution of the full protein structure (22, 23) and the absence of human pathogenic mutations reported for this gene. D174 and D175 are key for OGA’s catalytic activity (24), and the stalk domain has been shown to be critical for dimerization (22, 23). Interestingly, the Pseudo Histone acetyl transferase domain (pHAT) of OGA appears to be key in reading and binding histones (23).

To expand our mechanistic understanding of the link between *O*-GlcNAc enzyme variation and phenotype, we developed dedicated variant panels for both *OGT* and *OGA* that integrate all available information, including pathogenicity, cancer relevance, population frequency, and structural mapping. These pages provide a comprehensive, centralized resource for the study of *O*-GlcNAc enzyme variants.

For OGT, we identified a critical gap in ClinVar annotations, with several known OGT-XLID cases not properly represented. To resolve this, we implemented a direct submission portal within the *O*-GlcNAc database, enabling researchers and clinicians to contribute variant data for integration into the platform. This approach positions the *O*-GlcNAc database as the most current and comprehensive repository of OGT-XLID mutations (n = 101 OGT-XLID variants). Building on this framework, we also developed a dedicated patient-oriented page for OGT-XLID, which presents the mutation landscape alongside accessible explanations of OGT function and *O*-GlcNAcylation (oglcnac.mcw.edu/ogt_xlid/).

By consolidating *OGT* and *OGA* variant data into a one-stop resource, we were able to systematically analyze the variant landscape of each enzyme. This analysis revealed domain-specific enrichment of variants, identifying the most mutation-prone (“promiscuous”) regions in both proteins, as well as key mutations associated with improved (for OGT) or worsened (for OGA) cancer outcomes. Importantly, our bioinformatic analyses demonstrate that not all variants are created equal, underscoring the need for context-specific interpretation of OGT and OGA mutations in cancer.

Together, this work provides a comprehensive framework for interpreting *OGT* and *OGA* variants, offering new insights into their functional consequences and enabling more precise investigation of *O*-GlcNAc biology in human disease.

## Result

### *OGT* and *OGA* variants landscape

We created two dedicated pages for the *OGT* and *OGA* variants repository hosted in the *O*-GlcNAc Database. Variants were collected from ClinVar (25), GnomAD (26), and dbSNP (27) (Fig. 1*B*). A total of 4242 variants were cataloged for *OGT* and 3990 for *OGA*, distributed across genes, gene-flanking regulatory regions, 3′ and 5′ UTRs, Exons, splicing sites, and Introns (Fig. 1*C*). For each variant, we provided a separate page with associated publications, and consequences are provided as missense, synonymous, frameshift, intron, splice site variants, stop or start lost, and so on. Available information on the condition, as deposited on ClinVar, is also provided. Cross-referencing between ClinVar, dbSNP (rs ID), and GnomAD is also provided (Fig. 1C). We also created an automated search for literature references for each variant that appears at least twice in publications. Filtering and search functions are provided through a comprehensive top control panel, allowing users to explore variants by consequence, condition, or gene and protein changes. Variants can also be visualized and filtered across DNA, protein sequences, and protein structures (Fig. 1, *D* and *E*). We elected to use the AlphaFold-predicted structures of OGT and OGA (AF-O15294-F1, AF-O60502-4-F1) to map variants onto the protein models in the database, as these are the only structures that encompass the full-length proteins, including both structured and intrinsically disordered regions. This enables comprehensive visualization of all variants across the entire protein sequence, rather than restricting analysis to subsets covered by available crystallographic or cryo-EM structures.

### Integration of deposited and predicted variant pathogenicity

**Figure 2:**
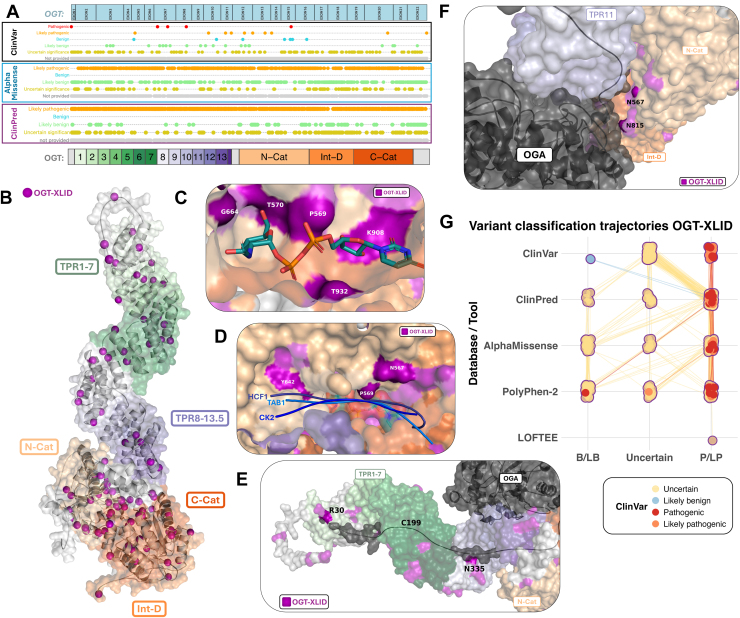
**Pathogenicity classification and structural mapping of *OGT* variants.***A*, linear representation of *OGT* variants across the protein sequence annotated with ClinVar, AlphaMissense, and ClinPred classifications. Domain organization is shown below. *B*, three-dimensional structural mapping of OGT-XLID patient (*purple*) variants onto the human OGT protein structure (AF-O15294-F1). *C*–*F*, structural close-up views highlighting OGT-XLID variants (*purple*) located within 4 Å of substrates or interaction partners. *C*, catalytic pocket with docked UDP-GlcNAc. *D*, catalytic pocket with docked peptide substrates (HCF1, TAB1, CK2). *E*, TPR domain interface showing docked OGA. *F*, OGT catalytic region (TPR11/N-Cat interface) in complex with OGA. *G*, Sankey-style plot showing variant classification trajectories for OGT-XLID variants, comparing original ClinVar annotations with predictions from ClinPred, AlphaMissense, PolyPhen-2, and LOFTEE after harmonization into benign/likely benign (B/LB), uncertain, and pathogenic/likely pathogenic (P/LP) categories.

We then wanted to present the pathogenicity proven or predicted for each variant, either clinically reported and deposited on ClinVar (25) or predicted based on sequence/structure analysis *via* Polyphen (28), AlphaMissense (29), ClinPred (30), and LoFTEE (31) tools (Table S1 and Fig. 2*A*).

We were first interested in comparing pathogenicity across databases and tools for each *OGT* variant, as OGT is known to be associated with OGT-XLID (9, 10, 11, 12, 13, 14, 15). To compare the various sources of pathogenicity for each variant, we collapsed the pathogenicity categories across resources into Pathogenic/likely pathogenic (P/LP), Benign/Likely Benign (B/LB), and Uncertain, as described in the methods. While almost all pathogenic variants on ClinVar were also categorized as P/PL in prediction tools, a significant number of variants classified as benign were predicted to be potentially pathogenic (Fig. S1*A*). We also noticed that 59 variants were reported in ClinVar for conditions associated with OGT-XLID, ID, or inborn genetic disease, but their pathogenicity remained uncertain, benign, or was not provided (Fig. S1*A*).

### Community-driven expansion and curation of the *O*-GlcNAc variant database

We identified previously unrepresented OGT-XLID patient variants that had been reported in prior presentations or publications but were absent from public repositories. To address this gap, we engaged with the research community to collect and incorporate these missing variants, resulting in a more comprehensive and up-to-date representation of OGT-XLID patient variants within the database (Fig. 2*B*). To ensure the continuity and maintenance of *O*-GlcNAc variants, we developed an online submission portal to facilitate community reporting of newly identified variants (Fig. S1*B*). This submission and curation workflow enables systematic incorporation of community-reported variants while preserving consistency with the existing annotation framework. By preventing duplication of existing entries and allowing structured updates to curated records, the system maintains continuity between user submissions and established database knowledge. Following validation, novel variants are fully integrated into the database and automatically processed through the complete annotation pipeline used for all entries. This includes cross-referencing with ClinVar, COSMIC (32), gnomAD, Genomic Data Commons (GDC) (33), PubTator, and additional functional annotation resources. As a result, newly submitted variants receive the same depth of annotation, interoperability, and visibility as variants derived from large-scale public repositories. Together, this workflow ensures that emerging clinically relevant variants, particularly those not yet captured in public databases, can be incorporated in a timely, curated, and standardized manner, reinforcing the *O*-GlcNAc Database as a living resource for rare disease and translational research.

### Disease-associated OGT-XLID variants localize to functional hotspots of OGT

To better observe the location of OGT-XLID variants, we performed molecular fitting between fitted multiple key molecules into this annotated OGT, including UDP-GlcNAc, various peptide substrate (HCF1 (34), CK2 (18), and TAB1 (35)) (Fig. S2*A*), and the *O*-GlcNAczyme complex that comprises an OGT dimer and OGA (36) (Fig. S2, *B* and *C*). Not surprisingly, we observed that a concentration of the symptomatic OGT-XLID variants is present in the catalytic pocket (C-Cat) where UDP-GlcNAc binds (Fig. 2*C*). Specifically, P569, T570 (2 OGT-XLID), G664, Y851, K908, and T932 were found to be within 4 Å of the UDP-GlcNAc (Fig. 2*C*). For the peptide substrate catalytic pocket, N567, P569, and Y642 were the XLID-identified residues within 4 Å of either of the protein substrates (CK2, TAB1, or HCF1) (Fig. 2*D*). For OGT/OGA interaction, R30, C199, and N335 were found to interact with OGA in the TPR groove, which is likely to be similar to other protein substrates. Similarly, N567 was also identified as one of the XLID residues at the interface, like the other protein substrate in the catalytic pocket (Fig. 2*E*). On the other hand, N815 was one of the XLID residues that seemed to be specific to OGT/OGA interaction (Fig. 2*F*). No XLID-specific residue was identified at the interface of OGT/OGT dimerization. No XLID-specific residue was identified at the nuclear localization sequence (NLS) (aa 461–463) (37), the D-BOX (aa 351/354) (38), or the PIP3 interaction surface (aa 991–992) (39).

### ClinPred provides the most accurate pathogenicity classification for OGT-XLID variants

Analysis of *OGT* variants’ pathogenicity showed that some predictive tools were more capable of predicting the demonstrated pathogenicity of OGT, with ClinPred capturing 84 OGT-XLID variants (n = 101) in the P/LP categories, while AlphaMissense captured 64, Polyphen-2 captured 52, and ClinVar captured only 13 (Fig. 2*G*). Therefore, we provide an option to view the ClinPred and the AlphaMissense prediction classification as well as the ClinVar on each variant page (Fig. 2*A*).

### Population allele frequencies reveal strong domain-specific constraint across OGT

**Figure 3:**
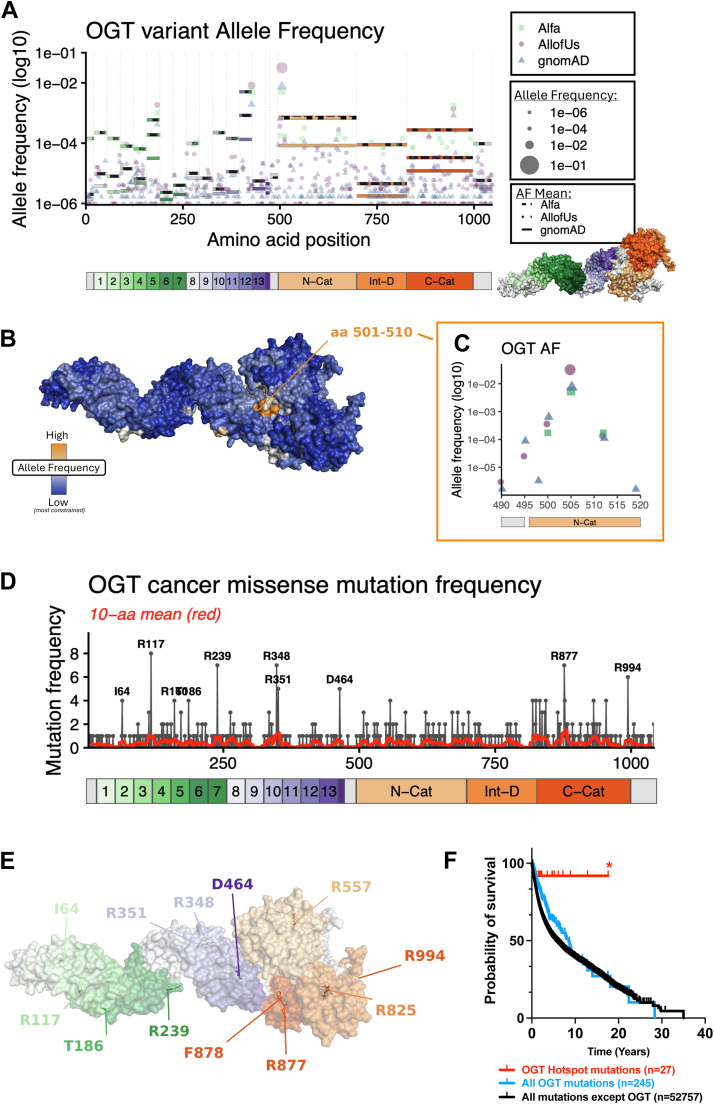
***OGT* variant allele frequency landscape and cancer-associated mutations.***A*, Allele frequency (AF) of *OGT* variants plotted along the protein sequence. Variants from gnomAD, All of Us, and ALFA are displayed, and domain-level AF means were computed for gnomAD and All of Us (insufficient data points for ALFA). *B*, structural representation of OGT colored according to the averaged allele frequency calculated in 10–amino acid bins and mapped onto the protein surface. *C*, zoomed view of residues 501 to 510 within the N-Cat domain, highlighting a region with comparatively elevated allele frequency. *D*, *OGT* cancer missense mutation frequency plotted across the protein sequence. In addition to individual mutation counts shown in *black*, the 10–amino acid sliding window mean is shown in *red*. *E*, three-dimensional structural mapping of *OGT* cancer hotspot mutations onto the human OGT structure. *F*, Kaplan–Meier overall survival curves comparing tumors harboring *OGT* hotspot mutations (*red*), tumors with all other *OGT* mutations (*blue*) and tumors with no *OGT* mutations (*black*). Statistical significance was assessed using the log-rank test (∗*p* < 0.05).

When available, allele frequency (AF) data were extracted from GnomAD (26), AllofUS (40), or NCBI ALFA (27) and displayed for each variant (Fig. S3*A*). In *OGT*, 2518 variants had allele frequency data (Fig. S3*B*). Across the full protein length, allele frequencies from gnomAD, All of Us, and the ALFA cohort are dominated by ultra-rare variants (≤10^−4^) (Fig. 3*A*), consistent with a strong purifying selection for OGT.

Domain-level analysis revealed a non-uniform distribution of allele frequencies. When AF values were averaged and normalized by domain length, several regions showed marked depletion of higher-frequency variants, suggesting increased functional constraint in these segments (TPR6, TPR10, TPR13 & intervening domain (Int-D)) (Fig. 3*A*). In contrast, TPR5, TPR12, and portions of the N-terminal and catalytic domains (N-cat, C-cat) exhibited relatively higher average allele frequencies, indicating comparatively relaxed tolerance to variation in specific subregions (Fig. 3*A*).

Importantly, allele frequency patterns were highly concordant between gnomAD and All of us, indicating that observed regional differences are robust across independent population cohorts (Fig. 3*A*). Given the extremely large sample size and predominantly disease-free composition of gnomAD, this dataset was used as the primary reference for structural AF mapping (Fig. 3*B*).

To assess spatial clustering of variation along the protein, allele frequencies were averaged in sliding 10–amino acid bins and mapped onto the three-dimensional structure of OGT (Fig. 3*B*). Consistent with strong overall constraint, only a single 10-amino acid bin (aa 501–511) displayed relatively elevated allele frequency (Fig. 3*B*). This enrichment reflects a localized hotspot of higher frequency variants within this short stretch (Fig. 3*C*). Notably, residue S510 has been reported in an XLID patient, although no population allele frequency is available at this position. In contrast, structurally and functionally critical regions, including the UDP-GlcNAc binding pocket Fig. S3*C*), the peptide-binding groove (Fig. S3*D*), and the OGT–OGA interaction interface (Fig. S3, *E* and *F*), mapped to some of the lowest observed allele frequencies, underscoring strong evolutionary constraint at catalytic and interaction surfaces essential for OGT function.

### *OGT* mutation hotspots in cancer associate with improved survival

For each variant in the database, we retrieved additional variants from the COSMIC (32) and GDC (33) databases and integrated them into the corresponding enzyme-specific pages (Fig. S4*A*). Some variants were already present in our database; thus, the total number of *OGT* variants increased to 4844 after integration. A total of 854 cancer samples (1.5% of the cBioportal sample set) harbored OGT mutations.

Among these, 736 *OGT* variants were associated with cancer and were distributed across the protein sequence (Fig. S4*B*). According to ClinPred classification, 331 *OGT* variants were categorized as likely pathogenic. Seventeen variants were identified in both cancer databases and the OGT-XLID dataset. Because COSMIC and GDC do not consistently report variant co-occurrence, causal relationships between individual *OGT* variants and specific cancer types cannot be definitively established.

Cancer-associated mutations were mapped onto the OGT sequence, and the ten most frequent mutations in cancer tissues were highlighted (Fig. 3*D*). By calculating mutation density over a 10-amino-acid sliding window, we identified regions with elevated mutation density, including the N-terminus (aa 1–10), TPR domains 5 to 8, and part of the N-Cat domain (aa 641–650) (Fig. S4*C*). In contrast, the UDP-GlcNAc binding site and the protein-binding groove exhibited among the lowest mutation densities and frequencies (Fig. S4, *D* and *E*). Mutation hotspots were also observed at the OGT–OGT interaction interface (L209) and the OGT–OGA interaction surfaces (R420 and P907) (Fig. S4, *F* and *G*).

To further evaluate hotspot relevance, we compared mutation frequency with allele frequency and identified residues with high mutation frequency but low allele frequency (Fig. S4*H*). These hotspots were annotated on the primary structure of OGT (Fig. 3*E*). The most relevant cancer-associated *OGT* mutations were E64, R117, E186, R239, R348, R351, D464, D553, K557, L825, N878, R877, and R994 (Fig. 3*E*). Several of these residues are located near the *OGT* nuclear localization signal (NLS; aa 461–463) or within the D-box region (aa 351–354). Notably, R348 has previously been identified as a methylation site. R117 and R994 have also been reported in XLID patients.

Kaplan–Meier survival analysis comparing patients harboring hotspot *OGT* mutations with those carrying other *OGT* mutations revealed improved survival in the hotspot group (Figs. 3*F* and S5*A*). This observation suggests that deleterious *OGT* mutations may confer a selective disadvantage to tumor progression.

Overall, *OGT* mutations, including the identified hotspots, were most frequently observed in common cancer types such as colorectal cancer, non-small cell lung cancer, melanoma, and endometrial cancer (Fig. S5, *C* and *D* and Table S2). However, hotspot mutations were depleted in melanoma, with only 1% of samples carrying hotspot mutations, compared to 12% for all *OGT* mutations (−11%) (Fig. S5, *C* and *D* and Table S2). In contrast, mature B-cell neoplasms exhibited enrichment of *OGT* hotspot mutations compared to other *OGT* mutations (+8%) (Fig. S5, *C* and *D* and Table S2).

A substantial proportion of *OGT*-mutated tumors exhibited concurrent alterations in additional cancer-associated genes (Table S3). Among genes mutated in at least 30% of tumors harboring *OGT* hotspot mutations, common tumor mutations such as TTN, MUC16, or P53 (Table S3) are present.

Additionally, 26% of *OGT*-mutant tumors also harbored mutations in POLE, a gene associated with ultra-hypermutated phenotypes. In addition, approximately 8% of *OGT*-mutant cases were classified as microsatellite instability (MSI)-positive. Together, these findings indicate that a subset of *OGT* mutations arose in tumors characterized by elevated mutational burden, consistent with hypermutated genomic contexts.

### Predicted pathogenic variants in OGA

To date, no *OGA* variants have been classified as pathogenic or likely pathogenic in ClinVar (Fig. S6*A*), indicating that definitive disease-causing mutations in *OGA* have not yet been established in curated clinical databases.

In contrast, multiple *OGA* variants are predicted to be deleterious by computational pathogenicity models, including ClinPred, and are distributed across all OGA domains (Fig. S6, *B* and *C*). To better understand the potential function of *OGA* variants, we fitted various structures to OGA, including p53 glycopeptide, the inhibitor Thiamet-G, and the OGA dimer structure (Fig. S7). Notably, a subset of these predicted deleterious variants localizes to the OGA–OGA and the glycopeptide interface (Fig. S6, *D* and *E*), suggesting that perturbation of substrate binding or dimerization may represent a potential mechanism of functional impairment.

### *OGA* allele frequency landscape reveals structural constraint at catalytic and dimerization interfaces

**Figure 4:**
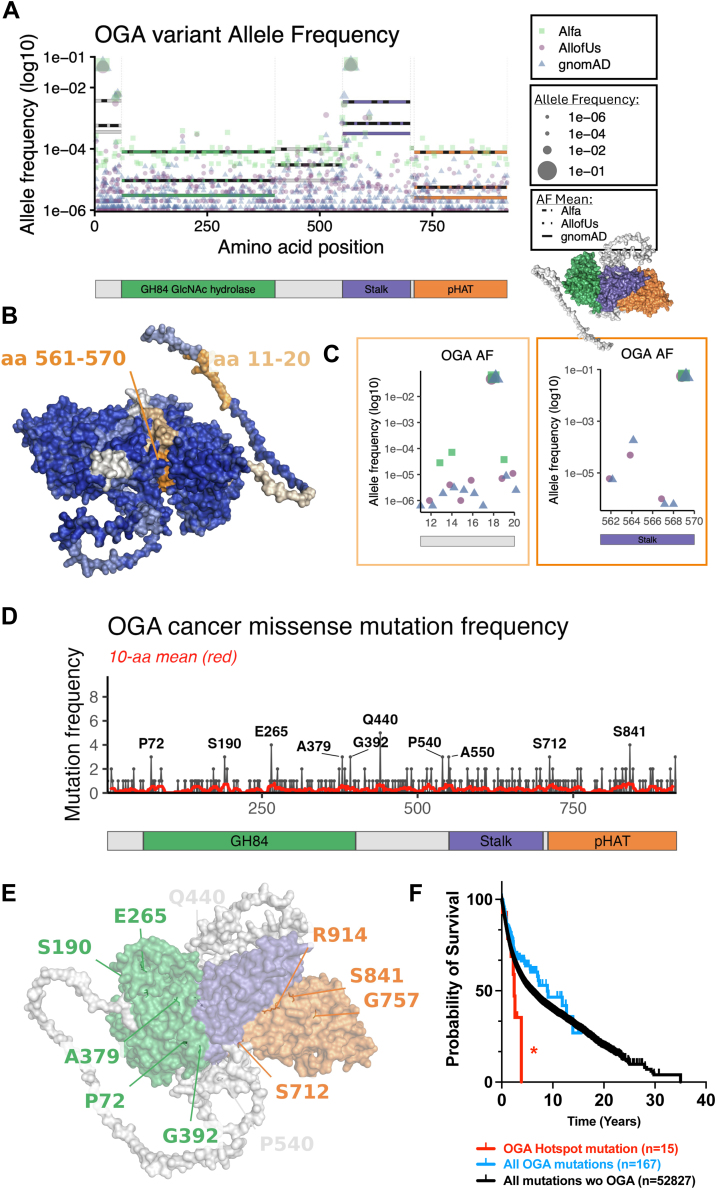
***OGA* variant allele frequency landscape and cancer-associated mutations.***A*, Allele frequency (AF) of *OGA* variants plotted along the protein sequence. Variants from gnomAD, All of Us, and ALFA cohorts are shown. Domain-level AF means were calculated for gnomAD and All of Us. *B*, Three-dimensional structural representation of OGA (AF-O60502-4-F1), highlighting the stalk region (aa 561–570) and N-terminal region (aa 11–20) for comparison of regional allele frequency patterns. *C*, zoomed views of allele frequency for residues 11 to 20 (*left*) and 561 to 570 (*right*), illustrating relative differences in population constraint across domains. *D*, *OGA* cancer missense mutation frequency plotted along the protein sequence. While individual mutation frequency is shown in *black*, the 10–amino acid sliding window mean is shown in *red*. *E*, three-dimensional structural mapping of *OGA* cancer hotspot mutations onto the human OGA structure. *F*, Kaplan–Meier overall survival curves comparing tumors harboring *OGA* hotspot mutations (*red*), tumors with other *OGA* mutations (*blue*) and tumors containing no *OGA* mutations (*black*). Statistical significance was assessed using the log-rank test (∗*p* < 0.05).

Across GnomAD, All of Us, and NCBI ALFA datasets, we identified 3070 *OGA* variants with available AF information (Fig. S3*B*). Variants were distributed across the entire coding sequence; however, the vast majority exhibited low allele frequencies (<10^−4^), consistent with rare or ultra-rare variation in the general population (Fig. 4*A*). This overall distribution suggests strong purifying selection acting on *OGA*.

When mapped across structural domains, higher-frequency variants were not randomly distributed (Fig. 4*B*). While most domains were depleted of higher-frequency variants, a relative enrichment of variants with modestly elevated AF was observed within the central stalk region. Zoomed analysis of residues 561 to 570 highlights this localized increase (Fig. 4*C*), contrasting with the N-terminal region (aa 11–20), which remains highly constrained.

Further structural mapping further clarified these constraints (Fig. S8). The glycopeptide-binding surface is highly conserved, with minimal tolerance for higher-frequency variation (Fig. S8*A*). Residues lining the substrate-binding cleft and catalytic pocket display uniformly low AF, consistent with their essential role in substrate recognition and hydrolysis. Similarly, the OGA–OGA dimerization interface is largely depleted of common variation, supporting the functional importance of dimer formation for structural stability and enzymatic activity (Fig. S8*B*). Notably, one residue at the dimer interface, Y569, lies within ∼4 Å of the opposing monomer and exhibits a comparatively higher allele frequency (Fig. S8*B*).

### OGA mutation hotspots in cancer associate with reduced survival

For OGA, a total of 505 variants were associated with cancers (Figs. S4*B* and 4*D*). A total of 469 cancer samples (0.8% of the cBioportal sample set) harbored *OGA* mutations. According to the ClinPred classification, 155 variants were likely pathogenic. Some variants were already present in our database; thus, the total number of *OGT* variants increased to 4358 *OGA* variants after integration.

Cancer-associated missense mutations were mapped along the OGA sequence and showed even distribution across the protein (Fig. 4*D*). Calculation of mutation density using a 10-amino-acid sliding window revealed regions of elevated mutation clustering across the catalytic domain, and at the junction of the stalk and the pHAT domain (Fig. S8*C*). Structural mapping of mutation frequency onto the OGA dimer demonstrated that residues forming the glycopeptide-binding surface exhibited comparatively low mutation frequency, with one residue, Y286, showing higher mutation frequency (Fig. S8*D*). Similarly, P2 showed relatively higher mutation frequency and was at the dimerization interface (Fig. S8*E*).

Integration of cancer mutation frequency with population allele frequency revealed that several recurrent cancer mutations occur at residues with low germline allele frequency, supporting their potential functional relevance (Fig. S8*F*). This includes residues S712, S841, G757, and R914 in the pHAT domain; residues P72, S190, E265, A379, and G391 in the catalytic domain; as well as Q440 and P540 in other undefined domains (Fig. 4*E*). Of these, S712 has been reported as a phosphorylation site (41).

Kaplan–Meier survival analysis comparing patients harboring hotspot *OGA* mutations to those with other *OGA* mutations demonstrated significantly reduced survival in the hotspot group (Fig. 4*F*). This was also true for the progression-free survival curve (Fig. S9*A*). This contrasts with observations for *OGT* and suggests that recurrent *OGA* mutations may promote tumor progression rather than impair it.

Overall, *OGA* mutations, including hotspot mutations, were most frequently observed in common solid tumors such as colorectal cancer, endometrial cancer, and non-small cell lung cancer (Fig. S9*B* and Table S4). However, hotspot mutations were relatively depleted in hepatobiliary cancer, endometrial cancer, and melanoma, indicating that *OGA* mutations in these cancers are more dispersed rather than recurrent at specific residues.

In contrast, several tumor types exhibited clear enrichment of hotspot mutations. The strongest enrichment was observed in non-small cell lung cancer (+8%) and esophagogastric cancer (+7%). These data indicate that *OGA* hotspot mutations show tumor-type–specific enrichment patterns rather than simply reflecting overall mutation frequency.

A substantial proportion of *OGA*-mutated cancer samples also harbored co-mutations in other genes, with some co-mutants (TTN, PTEN, TP53) representing more than 70% of the *OGA* cohort (Table S5). In addition, approximately 11% of *OGA*-mutant cases were classified as microsatellite instability (MSI)-positive.

In addition, 14% of *OGA*-mutated tumors also showed *OGT* mutations.

### Infrastructure modernization and user-facing improvements

As a result of the infrastructure modernization, the *O*-GlcNAc Database now operates on a fully updated, secure, and maintainable software stack, ensuring long-term sustainability of the resource. Backend upgrades improved application stability, performance, and compatibility with current and future dependencies, while the database upgrade supports more efficient data retrieval and scalability.

On the user-facing side, upgrading to Bootstrap v5.3.3 improved responsiveness, layout consistency, and cross-browser compatibility, enhancing accessibility across devices. Existing interactive features were preserved, and the introduction of Google Charts expanded visualization capabilities, complementing established tools such as D3.js, CanvasJS, and NGL Viewer. Together, these updates provide a more robust and extensible platform for data exploration and molecular visualization without disrupting existing workflows.

### Maintenance of variants pages

The variant pages of the *O*-GlcNAc Database are maintained through a scheduled bi-monthly update pipeline. Every two months, the database is refreshed with newly curated entries, during which newly reported variants from each of the referenced source databases are systematically retrieved, harmonized, and incorporated. This process ensures that *OGT* and *OGA* variant annotations remain current, while preserving consistent classification, metadata standards, and traceability across releases.

In addition to automated updates from external databases, the *O*-GlcNAc Database includes a community submission portal that allows direct submission of OGT-XLID variants that are not yet represented in ClinVar. This submission form enables clinicians and researchers to submit newly identified or unpublished variants, along with supporting information. Submitted variants undergo curation and annotation prior to public release, allowing timely incorporation of emerging clinical observations while maintaining data quality and consistency.

### Creation of the OGT-XLID page

**Figure 5:**
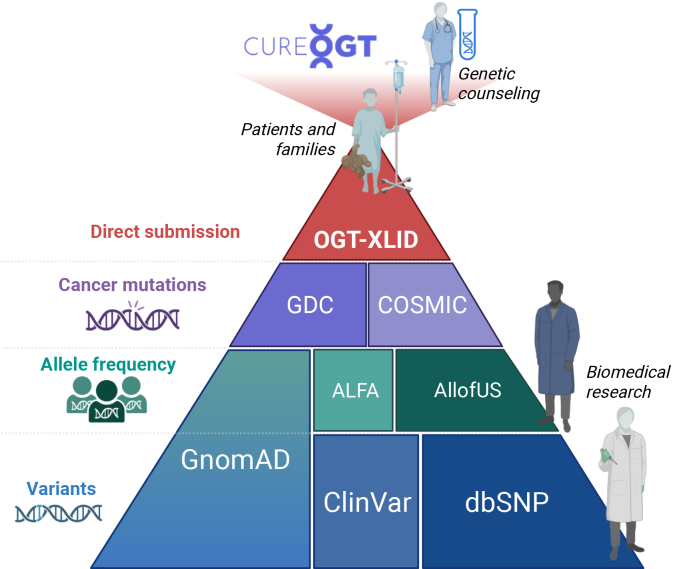
**Integrated framework linking public databases, cancer datasets, population variation, and community engagement within the *O*-GlcNAc Database.** Schematic overview illustrating the multi-layered architecture of the *O*-GlcNAc Database. The foundational layer integrates variants from public repositories (gnomAD, ClinVar, dbSNP). Cancer-associated variants from COSMIC and the Genomic Data Commons (GDC) are incorporated to provide somatic mutation context. Population allele frequency data from gnomAD, ALFA, and AllofUs refine variant interpretation. Disease-specific OGT-XLID variants are curated through direct community engagement. The *top* layer highlights the bidirectional connection between the database, patients and families, genetic counseling, and biomedical research, developed in partnership with the CureOGT Foundation.

To facilitate knowledge exchange and improve accessibility for all stakeholders, we developed a dedicated OGT-XLID page within the *O*-GlcNAc Database designed to bridge patients, clinicians, and researchers (Fig. 5). This page consolidates curated OGT-XLID variants, along with relevant molecular and clinical information, presented in a format accessible to patients and their families. Direct links to the variant pages and to the CureOGT Foundation website are provided, offering pathways to additional resources and support across patient, clinical, and research communities. Development of this resource was carried out in close collaboration with the CureOGT Foundation, ensuring that content addresses patient- and family-identified needs while maintaining scientific rigor.

## Discussion

With this update on the *O*-GlcNAc Database, we examined *O*-GlcNAc enzyme evolutionary constraints, developmental pathogenicity, and cancer-associated mutational patterns through a new lens. Only recently, with the identification of OGT-XLID arising from point mutations in *OGT*, have we come to appreciate how even subtle sequence alterations, particularly in non-catalytic domains, can have profound functional consequences (11, 13, 14, 20). Therefore, we created a one-stop resource to view all available information on *O*-GlcNAc enzyme variants. For each enzyme, we cross-reference variant databases (ClinVar, dbSNP, and GnomAD) with cancer mutation databases (COSMIC, GDC) and frequency allele datasets (GnomAD, AllofUs, and ALFA) (Fig. 5). The final result is provided in a digestible, easy-to-understand visual and searchable tool accessible on the *O*-GlcNAc database.

While putting this together, we noticed that some OGT-XLID patient variants that are often studied by *O*-GlcNAc research laboratories are not properly referenced online. Thus, we created an online submission platform to enable direct submissions from the research community and ensure the most up-to-date presentation of OGT-XLID mutations (n = 101 OGT-XLID variants). We also took the opportunity to develop a patient-oriented page for OGT-XLID, which provides the most current information on OGT-XLID mutations for patients, families, and clinicians. We hope that this serves as a guide to relevant research resources and connects the patient community. A direct link to the CureOGT Foundation website (https://cureogt.org/) provides access to educational materials, support networks, and opportunities for engagement across patient, clinical, and research domains.

### *OGT* and *OGA* pathogenicity landscape

Cross-referencing ClinVar and direct submissions with computational pathogenicity predictors (AlphaMissense, ClinPred, PolyPhen-2, and LoFTEE) enabled systematic annotation of variant pathogenic potential across *OGT* and *OGA*. Among these tools, ClinPred shows the highest concordance with known OGT-XLID patient mutations, suggesting strong performance in identifying clinically relevant *OGT* variants. Based on ClinPred classification, a substantial proportion of *OGT* variants are predicted to be deleterious, consistent with the strong evolutionary constraint observed across catalytically and structurally critical domains.

While no confirmed patients with pathogenic *OGA* mutations have been reported, ClinPred predicts multiple *OGA* variants as likely pathogenic, particularly those located in structurally important regions, including the OGA–OGA dimerization interface. Although these classifications rely on computational modeling, their enrichment at catalytic and interaction surfaces supports their potential functional impact.

Future identification of symptomatic *OGA* variants will be essential to benchmark predictive models and refine pathogenicity assessment for this gene. Collectively, these analyses suggest that, while *OGT* pathogenicity is clinically established, *OGA* may represent an underrecognized, structurally constrained candidate gene deserving further investigation.

### Coordinated evolutionary constraint across the *O*-GlcNAc cycling enzymes

Population-allele frequency analyses of both *OGT* and *OGA* reveal pronounced domain-specific evolutionary constraints, consistent with their key roles in human physiology. In *OGT*, higher-frequency variants are markedly depleted at catalytic, substrate-binding, and protein–protein interaction interfaces, including the UDP-GlcNAc binding pocket, peptide-binding groove, and OGT/OGA interaction surface, highlighting strong purifying selection at regions essential for enzymatic activity and regulatory coordination. Although discrete non-catalytic TPR segments show modest tolerance to variation, only a single localized hotspot (aa 501–511) emerges within an otherwise highly conserved protein. Similarly, *OGA* exhibits strong constraint at its catalytic region and dimerization interface, while portions of the N-terminal and stalk domains display comparatively relaxed tolerance. Together, these data indicate that both enzymes are subject to coordinated structural constraint, with evolutionary selection acting most strongly on residues required for substrate engagement, catalytic function, and maintenance of *O*-GlcNAc homeostasis.

### Opposing prognostic implications of recurrent *OGT* and *OGA* mutation hotspots in cancer

The identification of recurrent *OGT* mutation hotspots associated with improved overall survival suggests that a subset of *OGT* alterations impairs tumor fitness rather than promotes tumor progression. Although *OGT* cancer variants were broadly distributed across the protein, mutation density analysis revealed clustering within regulatory regions, including TPR domains and the N-Cat domain, while the UDP-GlcNAc binding pocket and protein-binding groove were comparatively protected from mutation. This selective depletion of mutations at catalytic and substrate-binding surfaces implies structural constraint and preservation of essential enzymatic function, consistent with OGT’s critical role in cellular viability. The observation that hotspot mutations occur near regulatory elements such as the nuclear localization sequence and D-box region further supports the possibility that these variants alter OGT localization or protein–protein interactions rather than abolish catalytic activity.

Comparison of cancer-associated and OGT-XLID substitutions at identical or adjacent residues revealed distinct biochemical patterns. At position 646, the XLID-associated A646T substitution introduces a polar uncharged residue, whereas the cancer-associated A646D variant introduces a negatively charged side chain. A similar contrast is observed at Y642, where a cancer-associated substitution to histidine introduces charge, while the XLID-associated Y642C substitution remains polar and uncharged.

These observations suggest that cancer-associated variants more frequently introduce charged residues at structurally relevant positions, potentially altering electrostatic interactions and protein–protein binding without fully destabilizing the enzyme. In contrast, XLID-associated substitutions may preferentially affect local stability or conformation. Together, this divergence implies distinct selective pressures acting on OGT in developmental *versus* tumorigenic contexts.

The improved survival observed in patients harboring *OGT* hotspot mutations contrasts with classical oncogenic drivers and instead raises the possibility that partial disruption of *O*-GlcNAc cycling may limit tumor growth or alter cellular stress adaptation. Importantly, 26% of OGT-mutant tumors also carried POLE mutations, and approximately 8% were MSI-positive, indicating that a subset of *OGT* mutations arises in hypermutated genomic contexts. While this suggests that some *OGT* variants may represent passenger events in ultra-mutated tumors, *OGT* mutations were not restricted to low-copy-number–altered, hypermutator phenotypes. Together, these findings support a model in which *OGT* mutations may be tolerated across multiple genomic instability backgrounds, with recurrent hotspot mutations potentially conferring functional effects improving clinical survival.

In contrast to *OGT*, recurrent *OGA* hotspot mutations were associated with significantly reduced overall and progression-free survival, suggesting a potential protumorigenic role for specific *OGA* alterations, as previously suggested (42). Although *OGA* mutations were distributed across the protein sequence, mutation density analysis identified clustering within the catalytic domain and at structural interfaces, including the dimerization surface and the pHAT domain. Integration of cancer mutation frequency with germline allele frequency revealed that several recurrent residues occur at positions rarely mutated in the general population, supporting their potential functional relevance. Notably, the relative protection of the glycopeptide-binding surface from mutation, with selective recurrence at specific residues such as Y286, implies functional constraint similar to that observed for OGT but with distinct structural targets.

OGA hotspot mutations are not observed within the stalk domain and, while overall associated with poorer clinical outcomes, suggest that impaired *O*-GlcNAc removal may enhance tumor fitness—potentially through sustained hyper-*O*-GlcNAcylation of oncogenic substrates—without disrupting OGA dimerization. This interpretation is also consistent with prior evidence linking elevated *O*-GlcNAcylation to tumor progression, metabolic adaptation, and stress resistance (43). It also aligns with a prior study that demonstrated that some OGA mutation (S652F) drives oncogenicity in cancer cell lines (42). In our hand, however, OGA hotspot enrichment in tumor types such as non-small cell lung cancer and esophagogastric cancer further supports tumor-type–specific selective pressures rather than random mutation accumulation. However, the frequent co-mutation of OGA with broadly mutated genes such as TTN, PTEN, and TP53, along with the presence of MSI-positive cases (∼11%), indicates that genomic instability also contributes to *OGA* mutation acquisition. Thus, while some *OGA* variants may arise in hypermutated contexts, the recurrence of specific low-allele frequency residues and their association with adverse survival argue for functional selection rather than purely passenger status.

In conclusion, we have developed an expanded and integrated resource focused on the two key enzymes of *O*-GlcNAcylation, *OGT* and *OGA*, providing a comprehensive, up-to-date variant landscape. By systematically integrating population and cancer datasets, we identified additional cancer-associated variants and characterized population-level constraint patterns, thereby refining our understanding of functional domains within OGT and OGA.

We also established a dynamic framework to maintain an updated OGT-XLID variant repository, helping to bridge a critical gap between patients, clinicians, and researchers.

We anticipate that this enhanced *O*-GlcNAc enzyme variant database will support improved diagnosis, facilitate research discovery, and promote informed, cross-disciplinary communication in both basic and translational settings.

Future directions include investigating partner enzymes that regulate UDP-GlcNAc substrate availability, as well as the salvage pathway (*e.g.*, NAGK), to provide a more complete understanding of how variation can impact *O*-GlcNAcylation.

## Experimental procedures

### System implementation and deployment

The *O*-GlcNAc Database infrastructure was updated to ensure long-term maintainability, security compliance, and compatibility with current software dependencies. The production environment was migrated to GNU/Linux (https://www.fsf.org/, https://www.kernel.org/) distribution Ubuntu 22.04.5 LTS (Jammy Jellyfish), replacing earlier versions. Core backend components and associated libraries were updated accordingly. Following the operating system migration, the backend framework was upgraded to Python v3.10.12 and Django v5.2.4 (44), ensuring compatibility with current Python packages and improving application stability and security. The database layer was updated to MongoDB v6.0.25 (45), enabling continued support for modern drivers and improved performance. The application was deployed using Nginx v1.18.0 (46) as a reverse proxy and static content server, with Gunicorn v21.2.0 (https://gunicorn.org) handling application-level request processing *via* the WSGI interface. HTTPS-only deployment standards were enforced using Transport Layer Security (TLS 1.2) and Server Name Indication. On the front end, the interface was built using the Bootstrap CSS framework (v5.3.3). Additional interactive and visualization components included jQuery (v3.7.1), CanvasJS, D3.js, Google Charts, and NGL Viewer (47) for dynamic data exploration and three-dimensional molecular structure visualization.

### Integration of variant data

Variant data were integrated to construct a comprehensive catalogue of single-nucleotide variants (SNVs) using the reference transcript ENST00000373719.8 for *OGT* and ENST00000361464.8 for *OGA*. Variants were retrieved from five primary resources: ClinVar (25), COSMIC (32), the GDC (33), dbSNP (27), and gnomAD (26). All datasets were harmonized using the GRCh38 human genome reference assembly. Insertions, deletions, and large structural variants were excluded, and only SNVs were retained. For gnomAD (v4) (26), variants were retrieved only if located within ±75 base pairs of annotated exons, reflecting current limitations of the gnomAD API. Each SNV was annotated using information collected from multiple APIs, including clinical significance, population-level allele frequencies, literature evidence, and predicted functional impact on the encoded protein.

### Clinical annotation

Clinical annotations were obtained from ClinVar (25), which aggregates expert- and laboratory-submitted interpretations linking genetic variants to human disease.

### Allele frequency annotation

Population allele frequencies were retrieved from gnomAD (v4) (26), All of Us (40), and NCBI ALFA (27). These complementary resources provide large-scale international sequencing data, a demographically representative US cohort, and aggregated multi-cohort frequency estimates, respectively. Together, they assign both global and population-specific allele frequencies to each variant.

### Cancer-associated mutations

Somatic variant information was integrated from COSMIC (v103) (32) and the GDC (33). COSMIC provides curated tumor-derived variants with tissue annotations and associated literature references, while GDC contributes variants identified across major cancer sequencing initiatives and links them to available clinical metadata. cBioPortal (48, 49) was utilized to navigate the combined cancer databases and extract mutations, survival, and case data. The datasets used for these analyses comprised 237 studies, representing a curated set of non-redundant studies with 97,547 samples.

### Functional impact prediction

Functional impact predictions were computed for all variants using Ensembl’s Variant Effect Predictor (VEP, latest release) (50). VEP was used to annotate each SNV with predicted molecular consequences at the transcript and protein levels. Pathogenicity scores, including Polyphen (28), AlphaMissense (29), ClinPred (30) and LoFTEE (31), were retrieved when available to provide a preliminary assessment of potential functional effects on OGT and OGA proteins.

### Literature referencing

Literature referencing was performed using the PubTator APIs (51). Each variant was queried using both cDNA-change and protein-change HGVS nomenclature, along with the corresponding gene symbol. Publications were retained only when a variant was mentioned in at least two independent references, thereby reducing noise from isolated or ambiguous reports.

### New OGT-XLID variant submission and curation workflow

To complement the automated update pipelines of the *O*-GlcNAc Database, we implemented a dedicated variant submission interface that allows users to report OGT-XLID variants not yet referenced in public repositories or not correctly annotated in ClinVar. The interface supports submissions derived from both research studies and patient-specific observations, while enforcing structured input requirements to enable downstream curation. Submitters are required to provide genomic coordinates, nucleotide and amino acid changes using HGVS nomenclature, the affected gene and transcript, and a valid contact email. These required fields ensure unambiguous variant identification and enable follow-up in cases of incomplete or inconsistent submissions. Upon submission, entries are transferred to an administrator-only review panel, where each variant appears as an annotated record. This panel displays user-provided information alongside automatically generated indicators, including whether the variant already exists in the database and its current review status. When a submitted variant corresponds to an existing database entry, the system retrieves and displays the relevant curated content. In such cases, no new variant record is created; instead, administrators may update the existing entry by incorporating additional clinical annotations or condition-specific tags (*e.g.*, OGT-XLID) provided by the submitter. For variants not previously recorded, the system initiates an automated annotation step using Ensembl VEP (50). VEP is used to verify concordance between the submitted genomic position, DNA change, protein consequence, and transcript identifier, and to generate predicted molecular consequences and transcript-specific annotations. Administrators manually review the VEP output against the submitted information prior to database integration.

### PyMOL structure visualization

Three-dimensional protein structure visualizations were generated using PyMOL (52). Human OGT and OGA structures were obtained from the Protein Data Bank (PDB) and used as structural templates for variant mapping and visualization. Specifically, the OGT structure was based on PDB ID: O15294-3 (AF-O15294-3-F1-v6), and the OGA structure was based on PDB ID: O60502-4 (AF-O60502-4-F1-v6).

Variants were mapped onto the corresponding protein structures using residue numbering aligned to the reference protein sequence. Structural representations were rendered using cartoon and surface representations, with variants highlighted using residue-level sticks and Cα spheres, as indicated in figure legends. All images were exported directly from PyMOL for figure assembly.

### Structural modeling and docking

Peptide- and UDP-GlcNAc-bound conformations obtained from published crystal structures were superimposed onto the canonical OGT model using Cα alignment in PyMOL. A similar approach was followed to align the OGT/OGA complex onto the OGT canonical model. All structural visualizations were performed in PyMOL. Interface residues were defined as amino acids containing at least one atom within 4 Å of the docked peptide or ligand. For OGT docking, the following alphaFold OGT (AF-O15294-F1) was used to fit the following Protein Data Bank (PDB) structures: UDP-GlcNAc (4GZ5), HCF1 (4N3B), CK2 (3PE4), TAB1 (4AY5), and OGT/OGA complex (7YEH). For OGA docking, the following alphaFold OGA (AF-O60502-4-F1) was used to fit the following PDB structures: glycosylated p53 (5UN8), OGA/OGA dimer (9NE2), and Thiamet-G (5UN9).

### Comparison of variant pathogenicity across databases and prediction tools

To enable consistent comparison of variant pathogenicity across heterogeneous databases and *in silico* prediction tools, pathogenicity categories were harmonized into three unified classes: pathogenic/likely pathogenic (P/LP), benign/likely benign (B/LB), and uncertain. For each source, original annotations or scores were collapsed as follows. For ClinVar (25), variants annotated as pathogenic or likely pathogenic were classified as P/LP, while benign and likely benign variants were grouped as B/LB. Variants annotated as variants of uncertain significance (VUS), conflicting interpretations, not provided, or related categories were classified as uncertain. For AlphaMissense (29), prediction scores were binned according to recommended thresholds: scores from 0 to 0.34 were classified as B/LB, scores from 0.34 to 0.564 as uncertain, and scores from 0.564 to 1 as P/LP. For PolyPhen-2 (28), variants annotated as benign were classified as B/LB, those labeled possibly damaging as uncertain, and those labeled probably damaging as P/LP. For ClinPred (30), predictions were categorized using either provided labels or score thresholds when applicable. Variants annotated as benign or with scores < 0.5 were classified as B/LB, those annotated as possibly pathogenic or with scores between 0.5 and 0.85 as uncertain, and those annotated as pathogenic or with scores > 0.85 as P/LP. For LOFTEE (31), high-confidence loss-of-function (LoF) variants were classified as P/LP, low-confidence LoF variants as uncertain, and filtered or non-LoF variants as B/LB.

### Data analysis and visualization

All data processing and statistical analyses were performed using R. Figures and schematic illustrations were generated using BioRender, GraphPad Prism and PyMOL was used to generate three-dimensional protein structure visualizations.

### Statistics

Overall survival curves were analyzed using Kaplan–Meier (KM) (53) method, and differences between groups were evaluated with the log-rank test (54) Hazard ratios (HR) and their 95% confidence intervals (CI) were calculated using Cox proportional hazards regression models (55). For pan-cancer analysis, cancer type was included in the Cow models to control for differences between tumor types and reduce potential bias due to tumor heterogeneity. All statistical tests were two-sided, and a *p*-value < 0.05 was considered statistically significant. When multiple gene-specific analyses were performed, *p*-values were corrected using the Benjamini-Hochberg false discovery rate (FDR) (56) method to limit the risk of false-positive findings.

Hotspot scores were computed for each residue using the following formula:

$$Hotspot_{i}=M_{i}\;\times \left(-\log_{10}\left(A\;F_{i}+\varepsilon \right)\right)$$

where M_*i*_ represents the number of mutations observed at residue *i* in the analyzed cohort, AF_*i*_ corresponds to the allele frequency of the same residue in the reference population dataset (GnomAD), and ε is a pseudo-count added to avoid undefined logarithmic values when AF_*i*_ = 0. In this study, ε was set to 1 × 10^−12^.

All analyses were performed in Python using the lifelines and statsmodels libraries (57, 58).

### Data availability

All data presented in this study are available through the *O*-GlcNAc Database at https://oglcnac.mcw.edu, with the version described here accessed on February 2026.

## Supporting information

This article contains supporting information.

## Conflict of interest

The authors declare the following financial interests/personal relationships, which may be considered as potential competing interests: S. O-V. S. is an editorial Board Member of JBC.
