# Supplementary material for: Systematic mapping of O-GlcNAc transferase and O-GlcNAcase defines disease-associated variants
Source: J Biol Chem. 2026 May 8;302(6):113134. doi: 10.1016/j.jbc.2026.113134 (PMC13264370; doi:10.1016/j.jbc.2026.113134)
Supplement: Supplementary Figures [file mmc1.pdf]

SUPPLEMENTARY FIGURES

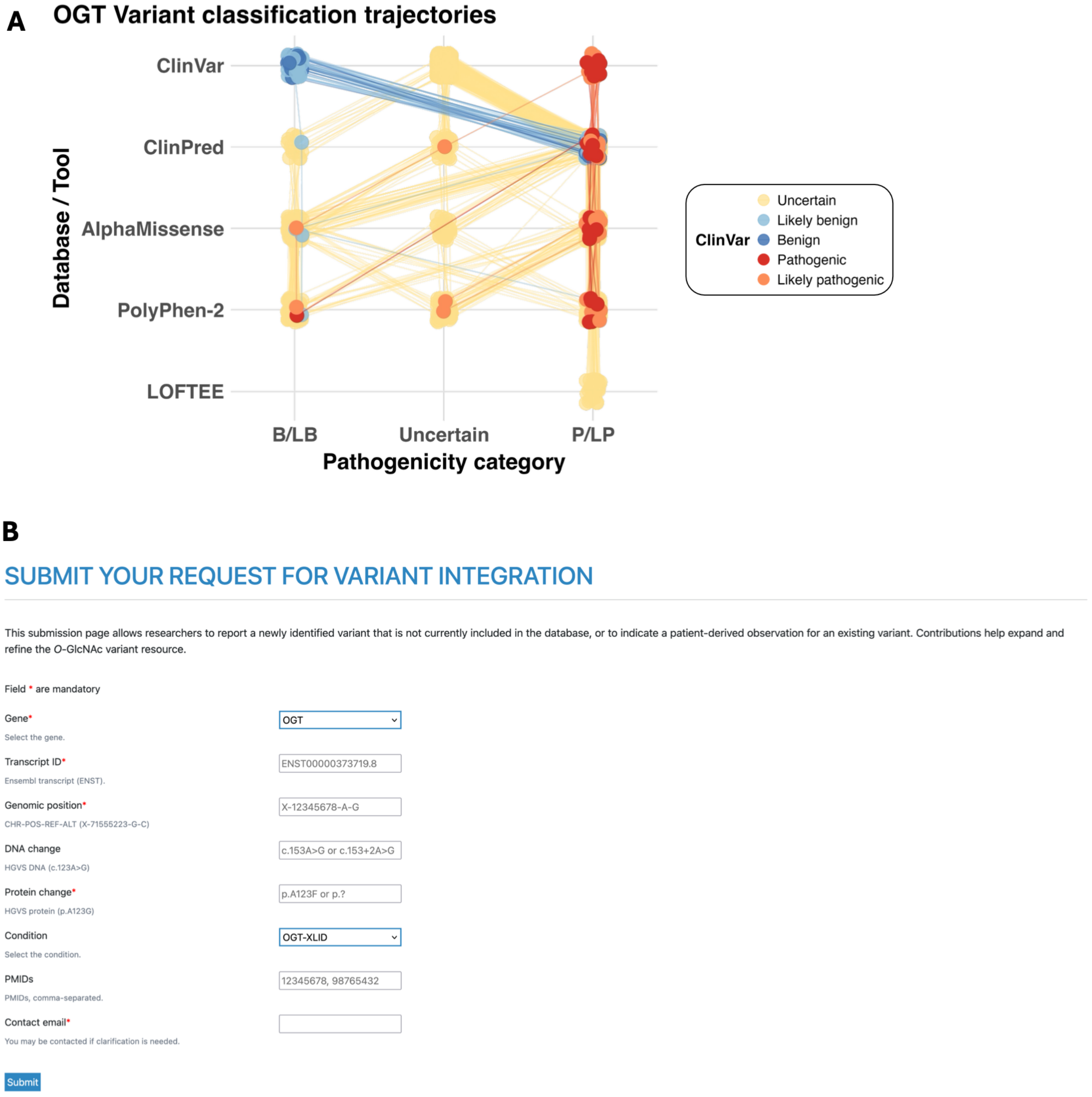

Figure S 1 : Variant pathogenicity harmonization and community-driven variant submission interface. (A) Sankey-style plot illustrating OGT variant classification trajectories across ClinVar and multiple in silico prediction tools (ClinPred, AlphaMissense, PolyPhen-2, and LOFTEE). Variants are harmonized into three categories—benign/likely benign (B/LB), uncertain significance, and pathogenic/likely pathogenic (P/LP). (B) Screenshot of the online variant submission interface within the O-GlcNAc Database. The submission form enables researchers and clinicians to report newly identified variants or patient-derived observations not represented in public repositories. Submitted entries undergo administrator review prior to curation and database integration.

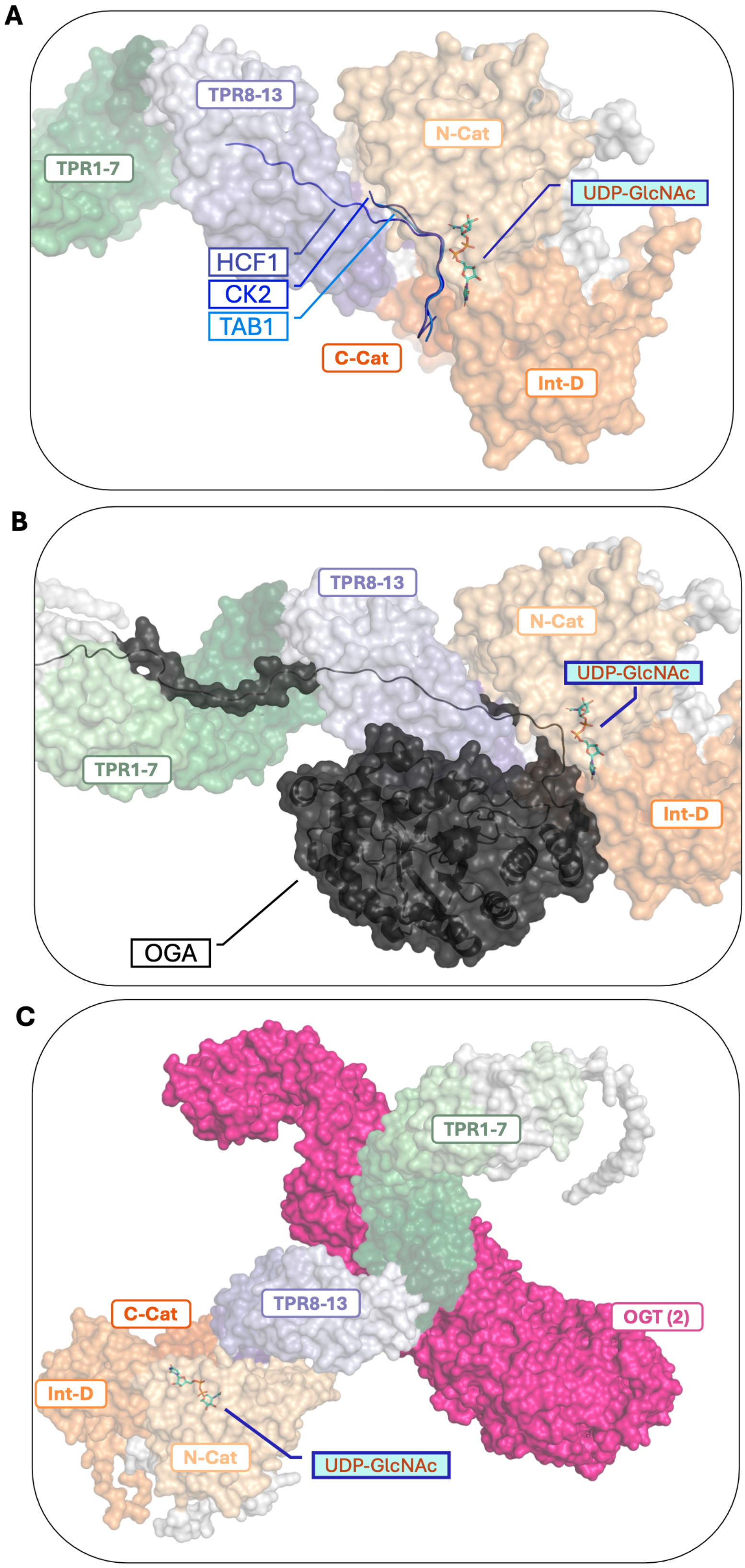

Figure S 2 : Structural fitting of OGT substrates and interaction partners. (A) Fitting of UDP-GlcNAc and three peptide substrates (CK2, HCF1, and TAB1) onto human OGT. (B) Fitting model of OGA onto OGT. (C) Fitting model of OGT dimer.

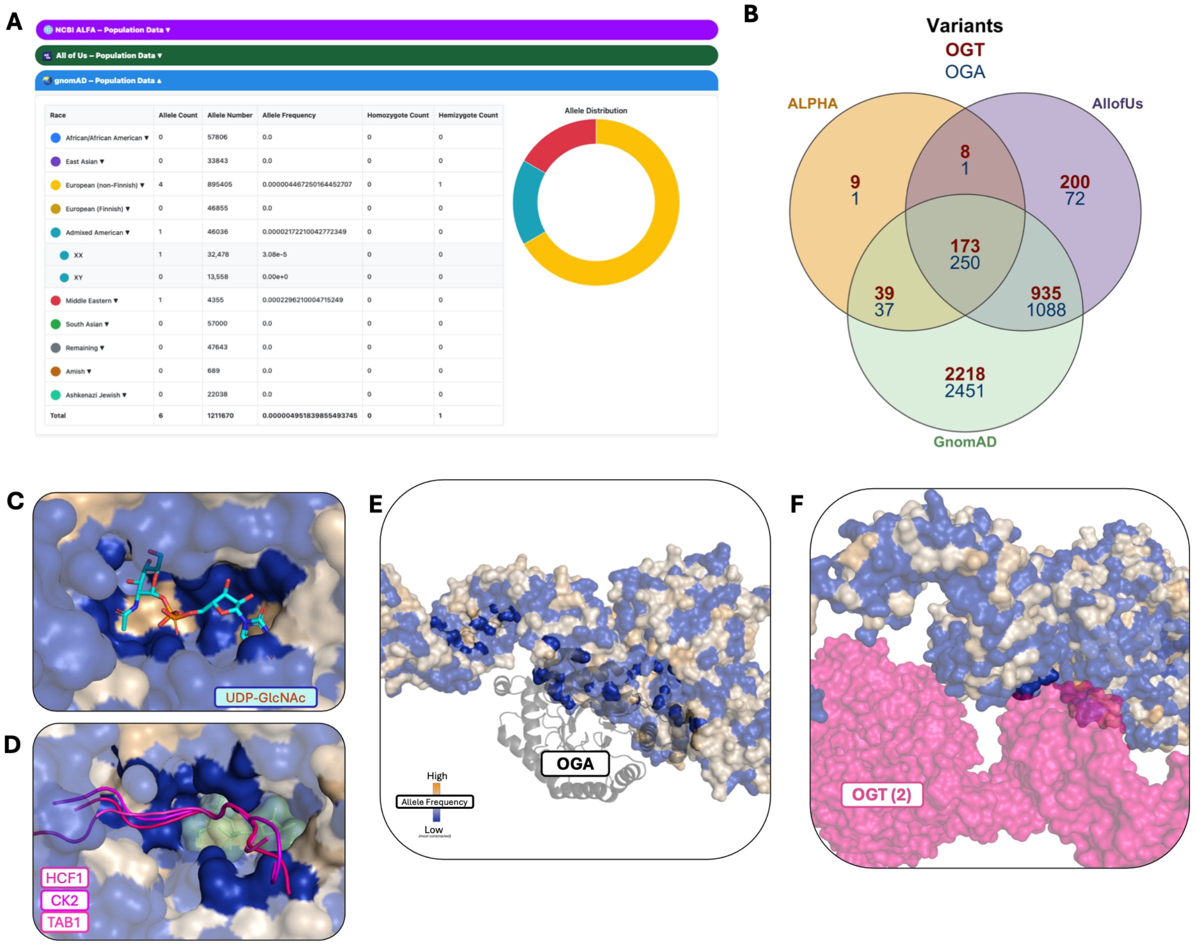

Race	Allele Count	Allele Number	Allele Frequency	Homozygote Count	Hemizygote Count
African/African American ▾	0	57806	0.0	0	0
East Asian ▾	0	33843	0.0	0	0
European (non-Finnish) ▾	4	895405	0.000004467250164452707	0	1
European (Finnish) ▾	0	46855	0.0	0	0
Admixed American ▾	1	46036	0.00002172210042772349	0	0
XX	1	32,478	3.08e-5	0	0
XY	0	13,558	0.00e+0	0	0
Middle Eastern ▾	1	4355	0.0002296210004715249	0	0
South Asian ▾	0	57000	0.0	0	0
Remaining ▾	0	47643	0.0	0	0
Amish ▾	0	689	0.0	0	0
Ashkenazi Jewish ▾	0	22038	0.0	0	0
Total	6	1211670	0.000004951839855493745	0	1

Figure S 3 : Population allele frequency integration and structural mapping of allele frequency constraints. (A) Representative view of the population frequency annotation panel for a single variant within the O-GlcNAc Database. The interface integrates allele frequency data from gnomAD, All of Us, and NCBI ALFA. (B) Venn diagram showing the overlap of OGT and OGA variants identified across gnomAD, All of Us, and ALFA cohorts. Variant counts for OGT (red) and OGA (blue) are shown within each dataset and intersection. (C–F) Structural mapping of allele frequency (AF) onto the surface of OGT. Residues within 4 Å of substrates or interaction partners are highlighted and colored according to AF (blue = low frequency/high constraint; orange = higher frequency). (C) Close-up view of the catalytic pocket with docked UDP-GlcNAc. (D) Close-up view of the catalytic pocket with docked peptide substrates (HCF1, TAB1, CK2). (E) OGT–OGA interaction interface showing AF distribution across the TPR region in complex with OGA. (F) OGT–OGT docking interface showing AF distribution near the interaction surface.

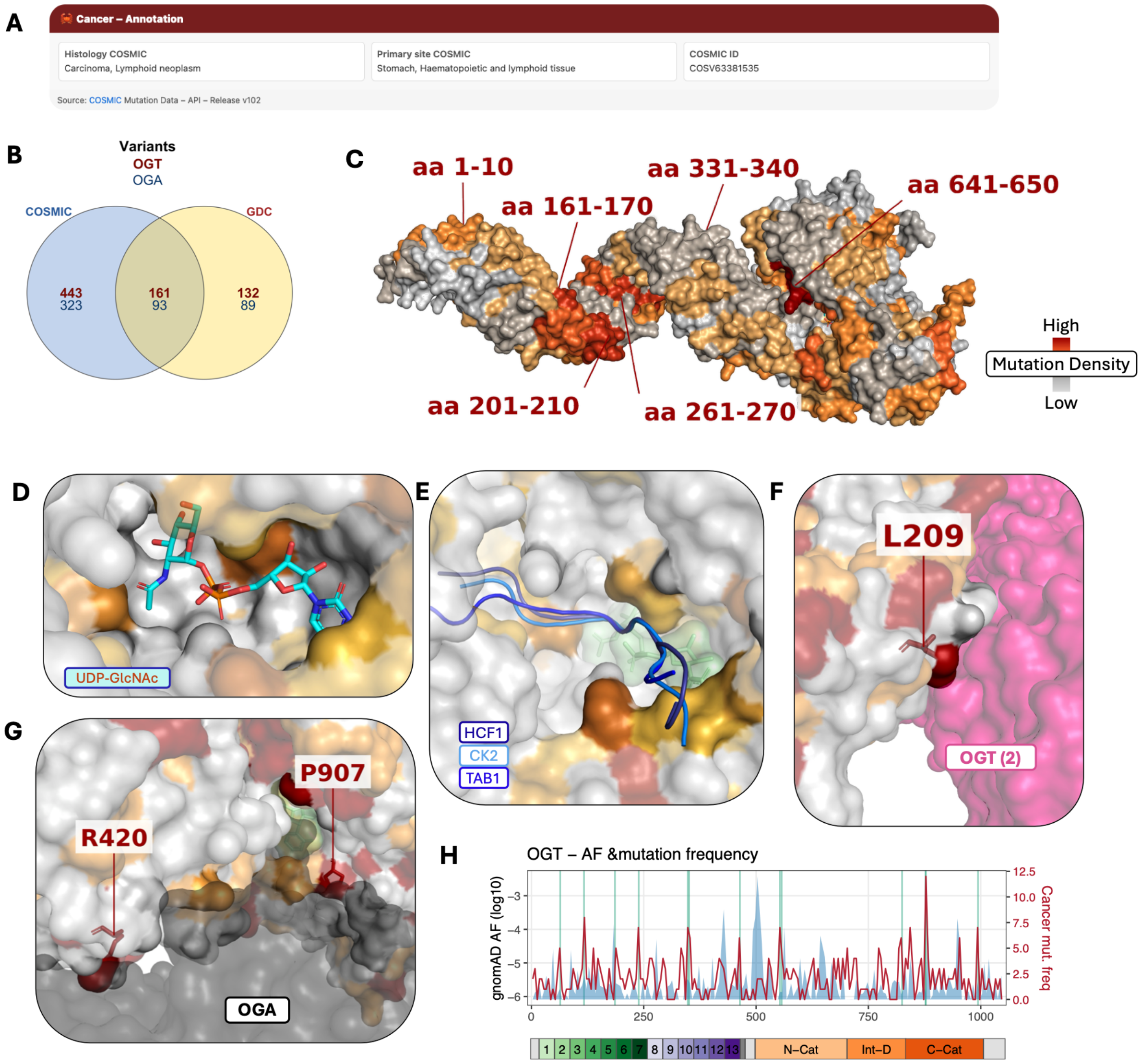

Figure S 4 : Cancer annotation and structural mapping of OGT somatic mutations. (A) Example of the cancer annotation module within the O-GlcNAc Database, illustrating display of somatic variant information from COSMIC. (B) Venn diagram showing the overlap of OGT and OGA variants identified in COSMIC and the Genomic Data Commons (GDC). Variant counts for OGT (red) and OGA (blue) are displayed within each dataset and intersection. (C) Structural mapping of mutation density (number of amino acids mutated per 10–amino acid bin) across OGT. Regions with the highest mutation density are labeled. (D–G) Structural mapping of cancer mutation frequency onto OGT surfaces within 4 Å of substrates or interaction partners. Residues are colored from gray (low mutation frequency) to red (high mutation frequency). (D) Catalytic pocket with docked UDP-GlcNAc. (E) Catalytic pocket with docked peptide substrates (HCF1, TAB1, CK2). (F) OGT–OGT interaction interface. (G) OGT–OGA interaction interface. (H) Overlay of population allele frequency (gnomAD AF) and cancer mutation frequency across the OGT sequence. Hotspot mutations are highlighted in green.

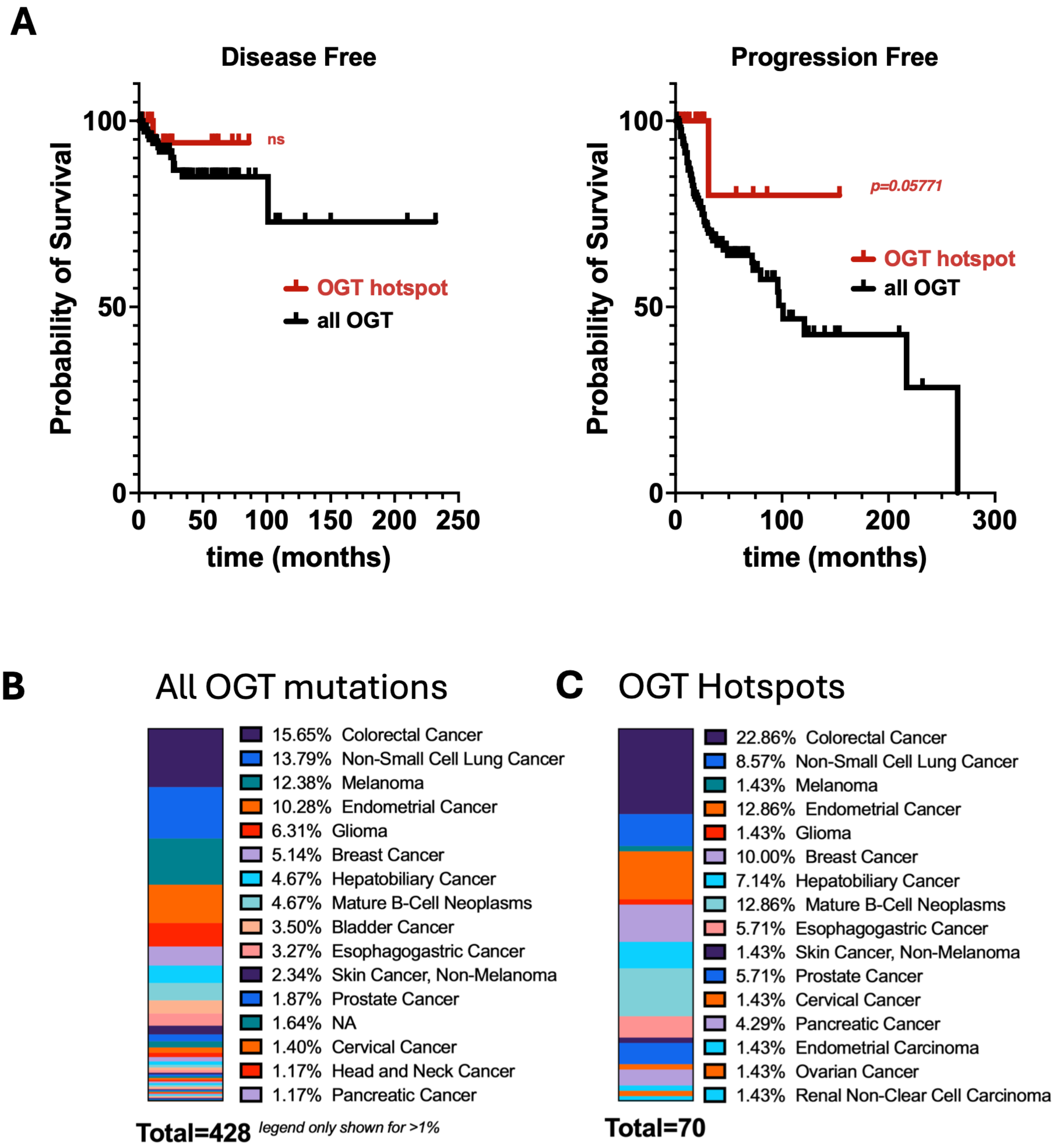

Figure S 5: Cancer type distribution and survival analysis in OGT-mutated tumors. (A) Kaplan–Meier survival curves derived from cBioPortal comparing tumors harboring OGT hotspot mutations (red) with tumors containing any OGT mutation (black). Disease-free survival (left) and progression-free survival (right) are shown. No significant difference was observed for disease-free survival (ns), while progression-free survival showed a trend toward improved outcome in hotspot-mutated tumors (p = 0.0577, log-rank test). (B) Distribution of cancer types among all OGT-mutated tumors. Percentages for tumor types representing >1% of cases are shown. (C) Distribution of cancer types among tumors harboring OGT hotspot mutations, highlighting differences in tumor-type representation compared to all OGT mutations.

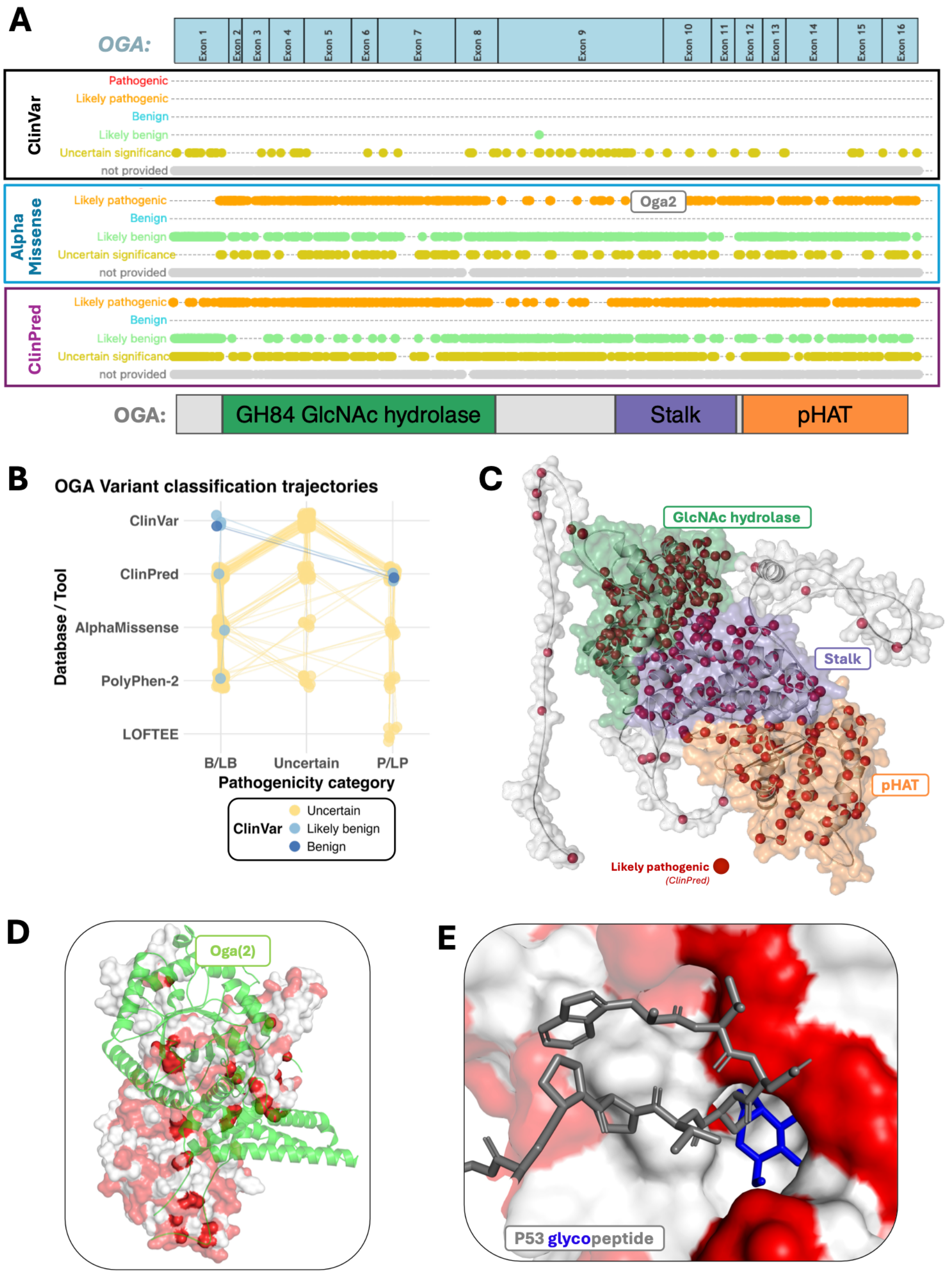

Figure S 6: Pathogenicity classification and structural mapping of OGA variants. (A) Linear representation of OGA variants across the protein sequence annotated with ClinVar, AlphaMissense, and ClinPred classifications. Variants are displayed according to amino acid position and color-coded by pathogenicity category for each source. (B) Sankey-style plot depicting OGA variant classification trajectories across ClinVar and in silico prediction tools (ClinPred, AlphaMissense, PolyPhen-2, and LOFTEE). Variants are harmonized into benign/likely benign (B/LB), uncertain significance, and pathogenic/likely pathogenic (P/LP) categories. (C) Three-dimensional structural mapping of OGA variants predicted to be likely pathogenic by ClinPred (red) onto the human OGA structure. (D) Structural representation of the OGA dimer highlighting ClinPred-predicted likely pathogenic residues (red) at or near the dimer interface. (E) Close-up view of the OGA–glycopeptide interaction interface (P53 glycopeptide), showing ClinPred-predicted likely pathogenic residues (red) relative to the substrate-binding surface.

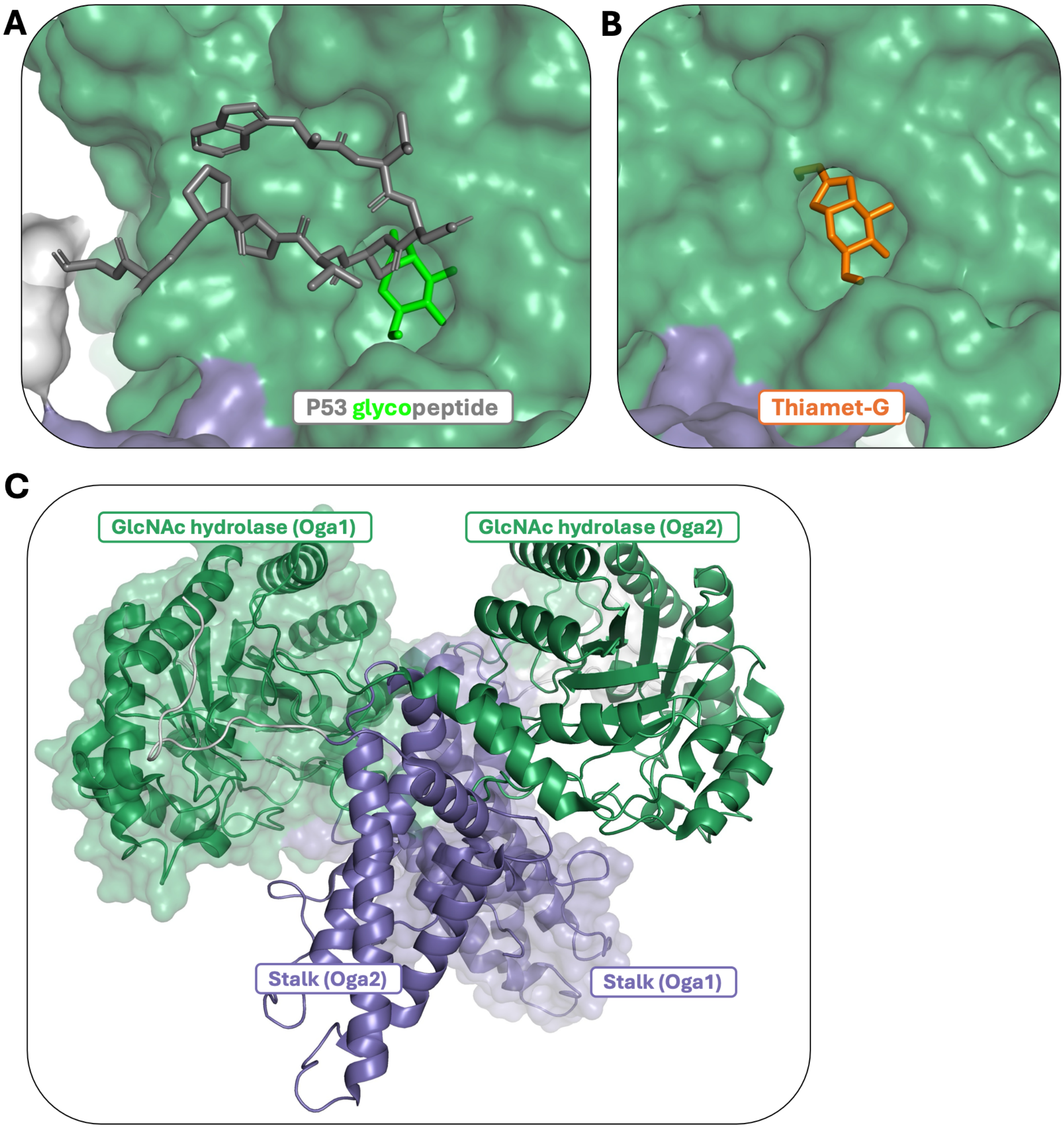

Figure S 7: Structural docking and dimer organization of OGA. (A) Docking model of a P53 glycopeptide substrate within the catalytic pocket of OGA. The glycopeptide is positioned within the GlcNAc hydrolase domain. (B) Docking model of the OGA inhibitor Thiamet-G within the catalytic pocket. (C) Structure of the OGA homodimer complex. The GlcNAc hydrolase domains (Oga1 and Oga2) and stalk regions are indicated, illustrating the dimer interface and overall domain organization

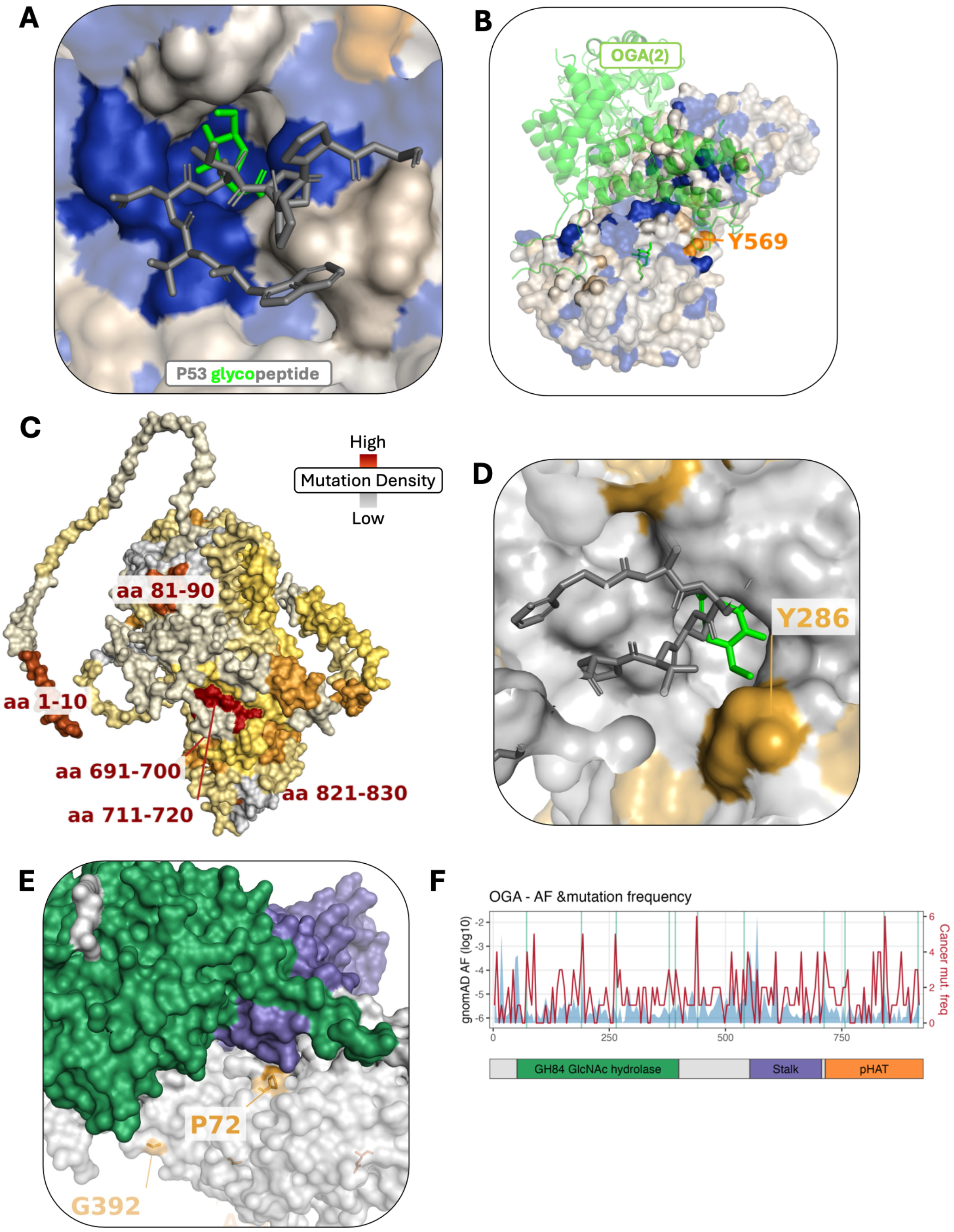

Figure S 8: Structural integration of OGA allele frequency and cancer mutation patterns. (A) Structural mapping of population allele frequency (AF) onto the OGA surface within 4 Å of the P53 glycopeptide-binding pocket. Residues are colored according to AF (blue = low frequency/high constraint; orange = high frequency). (B) Structural mapping of AF across the OGA dimer interface. (C) Structural representation of mutation density (number of mutated residues per 10–amino acid bin) mapped onto the OGA structure. (D) Close-up view of the catalytic pocket with docked p53 glycosylated, showing cancer mutation frequency. (E) Structural mapping of cancer frequency at the OGA dimer interface. (F) Overlay of population allele frequency (gnomAD AF, log10 scale) and cancer mutation frequency across the OGA sequence. Hotspot mutations are highlighted in green.

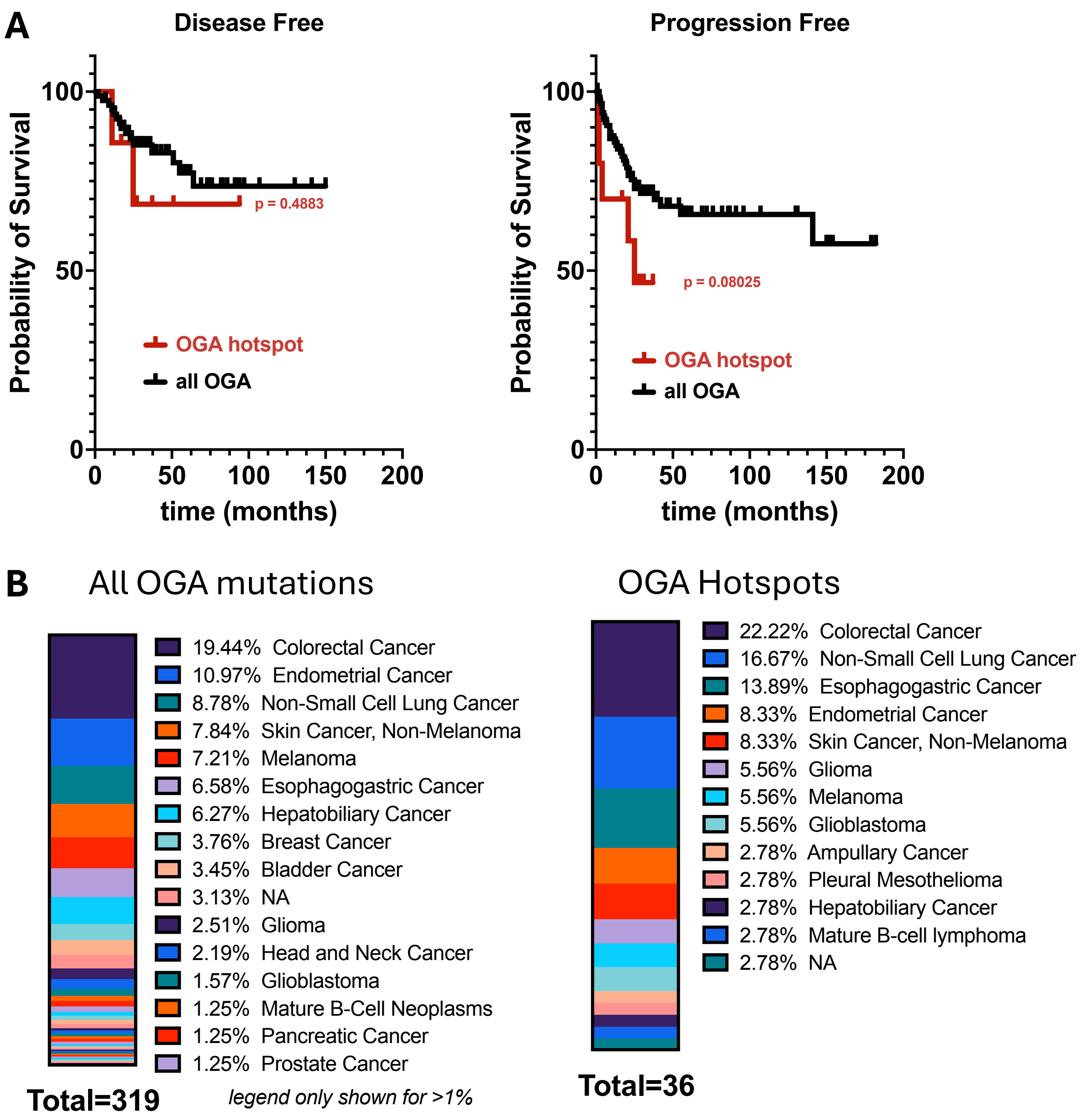

Figure S 9: Cancer-type distribution and survival analysis of OGA-mutated tumors. (A) Kaplan–Meier survival curves comparing tumors harboring OGA hotspot mutations (red) with tumors containing any OGA mutation (black). Disease-free survival (left) and progression-free survival (right) are shown (log-rank test). (B) Distribution of cancer types among all OGA-mutated tumors. Percentages for tumor types representing >1% of cases are shown. (C) Distribution of cancer types among tumors harboring OGA hotspot mutations, highlighting tumor-type enrichment relative to all OGA mutations.

SUPPLEMENTARY TABLES

Table S 1 : Databases and in silico prediction tools integrated into the O-GlcNAc Database. Summary of external variant databases and pathogenicity prediction tools incorporated for annotation and classification of OGT and OGA variants.

Table S 2 : Cancer type distribution of OGT-mutated tumors. Distribution and frequency of cancer types among tumors harboring OGT mutations, derived from cBioportal.

Table S 3 : Co-mutation landscape in OGT-mutated tumors. Recurrent co-mutated genes identified in tumors harboring OGT mutations, including mutation frequency and enrichment relative to background mutation rates derived from cBioportal.

Table S 4 : Cancer type distribution of OGA-mutated tumors. Distribution and frequency of cancer types among tumors harboring OGA mutations, derived from cBioportal.

Table S 5 : Co-mutation landscape in OGA-mutated tumors. Recurrent co-mutated genes identified in tumors harboring OGA mutations, including mutation frequency and enrichment relative to background mutation rates derived from cBioportal.
